# Smith County Medical Society

**Published:** 1904-03

**Authors:** A. Woldert

**Affiliations:** Secretary


### Society Notes.

---

### Smith County Medical Society.

---

The Smith County Medical Society met at the county court house in Tyler, Tuesday, February 9th, at 1:30 p. m., President Dr. Irvin Pope in the chair. The minutes of preceding meeting were read and approved.

Dr. Albert Woldert, of Tyler, read a paper entitled "The Importance of a Proper Diagnosis in the Treatment of Diseases of the Stomach."

Dr. W. S. Lacy, of Troupe, read a paper entitled "Malarial Hemoglobinuria." As to the treatment of this condition, he does not use quinine, and has not done so for years. He now resorts to diuretics, such as sweet spirits of nitre and acetate of potash. For the sick stomach he prefers to use applications of ice.

Dr. J. D. Phillips, of Tyler, always uses quinine. He makes it a custom to always give quinine to pernicious cases of malarial fever whether they suffer from hemoglobinuria or not. He believes that the first indication to be met in these cases is to control the paroxysms, and further to control the hemorrhage by the use of hemostatics, such as gallic acid, ergot and adrenalin chloride. He also uses diuretics.

In one case to which adrenalin had been given, and in which the hemorrhage had ceased, he subsequently gave quinine, but without the return of the hemoglobinuria. In the treatment of hemoglobinuria he could point to 90 per cent recoveries.

Dr. T. J. Bell, of Tyler, had never lost a case of malarial hemoglobinuria. He believed that if the disease was of malarial origin, and that if quinine was an antidote to malarial fever, then quinine should be given to these cases. In the treatment of this condition he believes it best to keep the stomach at rest, and hence prefers to give quinine hypodermically. As a diuretic he prefers to give calomel.

Dr. Albert Woldert, of Tyler, gave a short review of the history of this condition, and of the different views entertained by foreign investigators, especially to that of Koch, who maintained that the condition was brought on by the administration of quinine, and to that of Marchiafava and Bignami that it was due to a ferment in the blood, causing a rapid hemolysis, with consequent excretion in the urine.

Personally he had studied six cases of malarial hemoglobinuria, but had not had the opportunity of making a microscopic examination of the blood in all these cases. But of three cases, in which a protracted search had been made for malarial parasites, these protozoe had been found in two of these cases. In four of these cases, in which a careful examination of the urine had been made, granular casts and red-blood corpuscles had been found in three of them, while in one case neither was found. As to the question of the cause of hemoglobinuria occurring during the course of malarial fever, he pointed to the fact that the Cotton Belt Hospital, of Tyler, during the past sixteen years, had given to its patients many of whom (55 per cent) suffered from malarial fever) over 100 pounds of quinine without having ever produced a single case of hemoglobinuria.

The Board of Censors of the society then made a report of the eligibility to practice of every physician in Smith county, Texas, which was referred to the Committee on Public Health and Legislation. The society then adjourned to meet in Tyler the second Tuesday in March at 1:30 p. m.

A. Woldert, Secretary.

